# Childhood trauma and anger in adults with and without depressive and anxiety disorders

**DOI:** 10.1192/j.eurpsy.2023.270

**Published:** 2023-07-19

**Authors:** N. De Bles, L. E. Putz, N. Rius Ottenheim, A. M. van Hemert, B. M. Elzinga, B. W. Penninx, E. J. Giltay

**Affiliations:** 1 Department of Psychiatry, LUMC; 2 Department of Clinical Psychology, Leiden University, Leiden; 3 Department of Psychiatry, Amsterdam UMC/Vrije Universiteit, Amsterdam, Netherlands

## Abstract

**Introduction:**

Childhood trauma (CT) is associated with severe sequelae, including personality disorders and stress-related mental health disorders that can perpetuate long into adulthood.

**Objectives:**

We aimed to investigate (1) whether childhood trauma is associated with anger in adulthood, and, if so, (2) to explore which types of childhood trauma predominate in the prediction of anger, and (3) to explore whether the association is independent of psychopathology in a cohort that included participants without lifetime psychiatric disorders, with current or remitted depressive and anxiety disorders, or comorbid depressive and anxiety disorders.

**Methods:**

In the Netherlands Study of Depression and Anxiety (NESDA), childhood trauma was assessed with a semi-structured Childhood Trauma Interview (CTI) at baseline, and analyzed in relation to anger as measured at 4-year follow-up with the Spielberger Trait Anger Subscale (STAS), the Anger Attacks Questionnaire, and cluster B personality traits (i.e., borderline, antisocial) of the Personality Disorder Questionnaire 4 (PDQ-4), using analysis of covariance (ANCOVA) and multivariable logistic regression analyses. Post-hoc analyses comprised cross-sectional regression analyses, using the Childhood Trauma Questionnaire – Short Form (CTQ-SF) obtained at 4-year follow-up.

**Results:**

Participants (*n* = 2,276) were on average 42.1 years (*SD* = 13.1), and 66.3% were female. Childhood trauma showed a dose-response association with all anger constructs. Zooming in, all types of childhood trauma except for sexual abuse were associated with higher levels of trait anger, and a higher prevalence of anger attacks and antisocial personality traits in adulthood, independently of depression and anxiety. Additionally, all types of childhood trauma were significantly associated with borderline personality traits. Cross-sectionally, the effect sizes were larger compared to the analyses with the childhood trauma measured four years prior to the anger measures.

**Conclusions:**

Childhood trauma is linked with anger in adulthood, most strongly for trait anger and borderline personality traits. It is of clinical importance to explore childhood traumatic experience and start trauma-focused interventions when appropriate.

**Disclosure of Interest:**

None Declared

